# House dust mite induced allergic airway disease is attenuated in CD11c^cre^IL-4Rα^−/l^°^x^ mice

**DOI:** 10.1038/s41598-017-19060-9

**Published:** 2018-01-17

**Authors:** Natalie Eva Nieuwenhuizen, Frank Kirstein, Jennifer Claire Hoving, Frank Brombacher

**Affiliations:** 1 International Centre for Genetic Engineering and Biotechnology (ICGEB), Cape Town component, University of Cape Town, Cape Town, South Africa; 2 Institute of Infectious Disease and Molecular Medicine (IDM), Division of Immunology, University of Cape Town and South African Medical Research Council (SAMRC), Cape Town, South Africa; 3 Present Address: Department of Immunology, Max Planck Institute for Infection Biology, Chariteplatz 1, 10117 Berlin, Germany; 4 Present Address: Biovac Institute, 15 Alexandra Rd, Cape Town, 7405 Ndabeni South Africa

## Abstract

The precise mechanisms leading to development of T helper type (Th)2-driven allergic responses are unknown. We aimed to determine how IL-4 receptor alpha (IL-4Rα) signaling on CD11c^+^ cells influences allergen-induced Th2 responses in mice. CD11c^cre^IL-4Rα^−/l^°^x^ mice, deficient in IL-4Rα on dendritic cells and alveolar macrophages, were compared to IL-4Rα^−/l^°^x^ littermate controls in models of allergic airway disease induced by OVA/alum, OVA alone or house dust mite. Cytokine responses, eosinophil and neutrophil infiltration into the lungs, airway hyperreactivity and mucus hypersecretion were evaluated after allergen challenge. In the OVA/alum model, CD11c^cre^IL-4Rα^−/lox^ mice had similar airway hyperreactivity, eosinophil infiltration, Th2-type cytokine production and mucus hypersecretion to littermate controls. When alum was omitted during sensitization, CD11c^cre^IL-4Rα^−/lox^ mice had similar airway hyperreactivity and mucus secretion but reduced Th2-type cytokine production and eosinophils, suggesting alum overrides the requirement for IL-4Rα signaling on CD11c^+^ cells in enhancing Th2-type responses. In the house dust mite model, CD11c^cre^IL-4Rα^−/lox^ mice showed similar mucus secretion, but reduced Th2 responses, eosinophils, neutrophils and airway hyperreactivity, unlike previously tested LysM^cre^IL-4Rα^−/lox^ mice, which lack IL-4Rα on alveolar macrophages but not on dendritic cells. Therefore, our results indicate that IL-4Rα signaling on dendritic cells promotes allergen-induced Th2 responses and eosinophil infiltration into the lung.

## Introduction

Adaptive cellular immune responses, including T cell responses, are driven by professional antigen presenting cells. Dendritic cells (DCs), the sentinels of the immune system, can present antigen in an immunogenic or a tolerogenic manner in order to elicit the appropriate adaptive response. While the signals that drive DC driven induction of Th1 and Th17 responses are well understood, the mechanisms leading to the Th2 differentiation observed in allergic disease and helminth infection are still unclear^1^. However it is well-established that IL-4 plays an important role^1,2^, and in the absence of DCs, Th2 responses are dramatically reduced^3^. Recently it was shown that IL-4 treatment of DCs results in upregulation of markers typically associated with alternatively activated macrophages, including RELM-α, which appeared to be important in promoting optimal Th2 responses^4^.

While IL-4 is the primary inducer of Th2 responses^5^, paradoxically it has also been shown that IL-4 promotes IL-12 production by bone marrow derived dendritic cells (BMDCs) stimulated with CpG or LPS by inhibiting production of regulatory IL-10^6–9^. In a recent publication, we demonstrated that IL-4Rα signaling on DCs was required for optimal Th1 responses in an *in vivo* model of *Leishmania major* infection by generating CD11c^cre^IL-4Rα^−/lox^ mice, which lack IL-4Rα signaling on DCs^10^. Furthermore, IL-4Rα signaling was required to avoid tissue damage after *Leishmania* infection following DC-mediated vaccination of BALB/c mice^11^. IL-4Rα can associate with either the common gamma chain to form IL-4 receptor 1, through which only IL-4 is able to signal, or with IL-13Rα1, forming IL-4 receptor 2 through which both IL-4 and IL-13 can signal. CD11c^cre^IL-4Rα^−/lox^ mice demonstrated hypersusceptbility to infection, associated with decreased IL-12 and Th1 responses and reduced killing effector functions of myeloid DCs.

Since both IL-4 and DCs are required for optimal Th2 responses, we aimed to determine how loss of IL-4Rα signaling on DCs would affect adaptive immune responses in Th2 driven pathologies such as allergic airway disease. We found that Th2 responses were impaired in the absence of DC IL-4Rα signaling, although alum adjuvant could override the requirement for DC IL-4Rα signaling in generation of Th2 responses. The absence of IL-4Rα signaling on DCs significantly reduced eosinophils, neutrophils and airway hyperreactivity (AHR) in house dust mite-induced allergic airway disease, showing that IL-4 acts directly on DCs to exacerbate allergic airway disease.

## Methods

### Generation and genotyping of CD11c^cre^IL-4Rα^−/lox^ BALB/c mice

CD11c^cre^ C57/BL6 mice^12^ were backcrossed to BALB/c for at least 9 generations, crossed with IL-4Rα^−/−^ BALB/c mice^13^ to establish CD11c^cre^IL-4Rα^−/−^ BALB/c mice^10^ then intercrossed with IL-4Rα^lox/lox^ BALB/c mice^14^ to generate hemizygous CD11c^cre^IL-4Rα^−/lox^ mice. This strategy avoids possible aberrant early loxP excision and increases Cre efficiency due to reducing substrate (loxP) by 50%. Hemizygous littermate controls (IL-4Rα^−/lox^) were used as controls in all experiments. Mice were genotyped as described previously^14^. All mice were housed in specific-pathogen free barrier conditions in individually ventilated cages. Experimental mice were age and sex matched and used between 8–12 weeks of age.

### Ethics statement

This study was performed in strict accordance with the recommendations of the South African national guidelines and University of Cape Town practice for laboratory animal procedures. All mouse experiments were performed according to protocols (011–032 and 011–029) approved by the Animal Research Ethics Committee of the Health Sciences Faculty, University of Cape Town and all efforts were made to minimize discomfort of the animals.

### Mouse models of allergic airway disease

For ovalbumin (OVA)-alum induced allergic airway disease, mice were sensitized intraperitoneally with 50 μg OVA (Sigma-Aldrich, grade V) in 200 μl PBS/1.3% alum (Sigma Aldrich) or PBS/alum as a control, on day 0, 7 and 14, as described previously^15–17^. On day 21, 22 and 23, mice were challenged intranasally under anaesthetic with 1 mg OVA in 50 μl PBS, or PBS as a control. AHR and immune responses were measured on day 24. For alum-free OVA-induced allergic airway disease the same protocol was followed, with the omission of the alum. For house dust mite (HDM)-induced allergic airway disease, mice were anaesthetized and sensitized intranasally with 100 μg HDM extract (Greer Laboratories, Lenoir, U.S.A.) on day 0 and 10 μg HDM extract on days 7 to 11, as previously described^1^. AHR and immune responses were measured on day 14.

### Airway hyperreactivity

Airway resistance and elastance of the whole respiratory system (airways, lung chest wall) after challenge with increasing doses of acetyl-β-methylcholine (methacholine, Sigma-Aldrich) was determined by forced oscillation measurements as previously described^18^ with a Flexivent system (SCIREQ, Montreal, Canada) using the single-compartment (“snapshot”) perturbation. Differences in the dose-response curves were analyzed by repeated-measures ANOVA. Only mice with acceptable measurements for all doses (coefficient of determination > 0.95) were included in the analysis. After the procedure, mice were killed and tissue samples were taken for analysis.

### Histology

Lungs were fixed in 4% formaldehyde in phosphate buffered saline and embedded in wax. Tissue sections were stained with Periodic-acid-Schiff. The histological mucus index (HMI) was used to quantify pulmonary goblet cell hyperplasia in individual mice. Airways were photographed at a magnification of 20× (2× objective) and overlaid with a standard grid of 5 mm in Photoshop. The number of mucus-positive squares was presented as a percentage total number of squares with epithelial cells to determine the Histological Mucous Index (HMI) as previously described^19^.

### Flow cytometry

The following antibodies were used for flow cytometry: SiglecF-PE, CD11c-APC, CD11b-biotin, GR-1-FITC, CD3-FITC, CD4-PerCP, CD8-biotin, (all BD Bioscience. Erembodegem, Belgium except GR-1-FITC, made in-house) and PerCP or APC streptavidin (BD Bioscience). Eosinophils were CD11b^+^SiglecF^+^CD11c^−/int^GR-1^int^. Neutrophils were CD11b^+^GR-1^high^CD11c^−^SiglecF^−^. For intracellular cytokine staining, lung cells were seeded at 2 × 10^6^ cells/well and stimulated at 37 ^o^C for 4 hours with phorbal myristate acetate (Sigma-Aldrich) (50ng/ml), ionomycin (Sigma-Aldrich) (250ng/ml) and monensin (Sigma-Aldrich) (200 μM) in DMEM/10% FCS. CD4+ T cells were stained with CD3-biotin with APC labeled streptavidin and CD4 PerCP, fixed and permeabilized, and intracellular cytokines were stained with PE anti-cytokine antibodies and isotype controls (BD Bioscience). Cells were acquired on a FACS Calibur machine (BD Immunocytometry systems, San Jose, CA, USA) and data were analyzed using Flowjo software (Treestar, Ashland, OR, USA).

### Antigen-specific restimulation

Mediastinal lymph node cells were isolated by pressing through 70  μM cell-strainers, red blood cell lysis was performed and white blood cells were washed and resuspended in 10% IMDM (Gibco). Whole lymph node cells were restimulated with anti-CD3 (100 μg/ml), house dust mite extract (100 μg/ml), OVA (100 ug/ml) or medium control^1^. After 72 h incubation at 37 ^o^C, supernatants were collected and cytokine production was analysed as previously described.

### ELISAs

Cytokines in cell supernatants were measured by sandwich ELISA as previously described^20^. For antibody ELISAs, blood was collected in serum separator tubes (BD Bioscience, San Diego, CA). Antigen-specific IgG1, IgG2a, IgG2b and IgE were quantified by ELISA, as previously described^20^.

### T cell differentiation and proliferation assays

CD4^+^ T cells were positively selected from lymph nodes of naïve mice ( > 95% purity). Cells were used for *in vitro* Th1/Th2 differentiation assays and proliferation assays as previously described^13,21^. Briefly, for proliferation assays, cells were stimulated with serially diluted IL-4 or IL-2 for 48 hours at 37 ^o^C, then pulsed with 1 μCi thymidine (AEC Amersham Uppsala, Sweden) for 18 hr. Thymidine incorporation was measured in a liquid scintillation counter. For differentiation assays, cells were incubated with anti-CD3 and anti-CD28 and Th1/Th2 differentiation was stimulated by IL-12/anti-IL-4 or IL-4/anti-IFN-γ, respectively. IL-4 and IFN-γ were measured in supernatants.

### Statistics

Data is given as mean ± SEM. Statistical analysis was performed using the unpaired 1-way Anova with Tukey’s post test, defining differences to IL-4Rα^−/lox^ PBS mice as significant (*, p ≤ 0.05; **, *p* ≤ 0.01; ***, p ≤ 0.001) unless otherwise indicated by brackets. (Prism software; http://www.prism-software.com).

### Data availability

Data generated during and/or analysed during the current study are included in this published article and are available from the corresponding author on reasonable request.

## Results

### Loss of IL-4Rα signaling on CD11c^+^ cells leads to attenuated allergic airway disease in the absence of alum adjuvant

**Figure 1:**
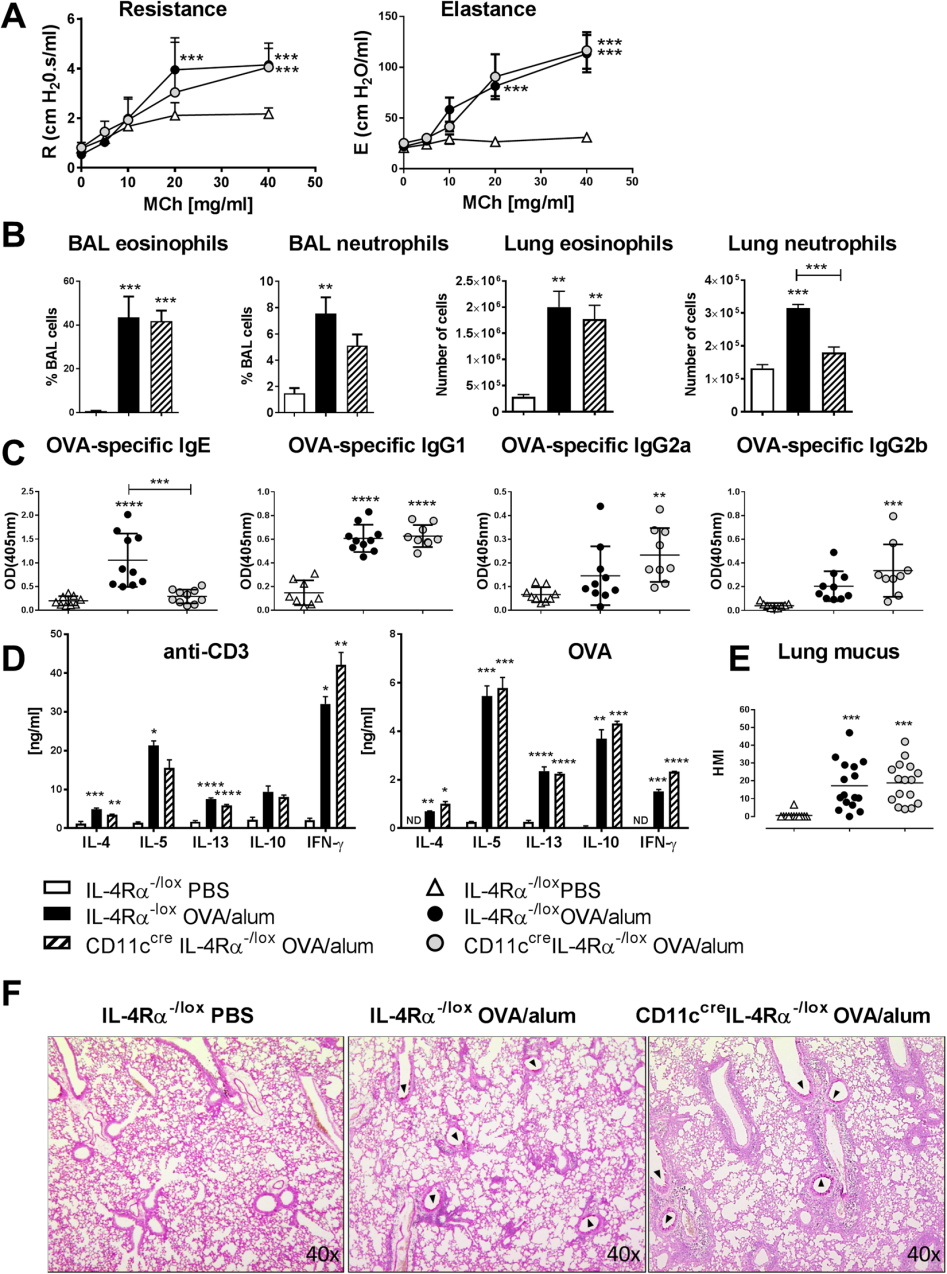
Allergic airway disease in CD11c^cre^IL-4Rα^−/lox^ mice in the OVA-alum model (day 24). (**A**) Airway resistance and elastance after methacholine challenge (**B**) Eosinophils and neutrophils in the airways and lungs of challenged mice. (**C**) OVA-specific antibodies measured in the serum. (**D**) Cytokines measured by ELISA after restimulation of draining lymph node cells with anti-CD3 or OVA. (**E**) Goblet cell hyperplasia. HMI = histology mucous index. (**F**) Mucus hypersecretion in the airways (100× magnification). All graphs are representative of 2 experiments, with n = 5–6 per experiment. Significant differences are shown between IL-4Rα^−/lox^ PBS and the other groups unless otherwise indicated by brackets. *P < 0.05; **P < 0.01; ***P < 0.001; ****P < 0.0001.

Previously we used LysM^cre^IL-4Rα^−/lox^ mice in both OVA- and HDM-induced models of allergic airway disease, and did not find a role for IL-4Rα signaling on macrophages, which included alveolar macrophages^18^. We have shown that CD11c^cre^IL-4Rα^−/lox^ mice lack IL-4Rα expression on DCs and alveolar macrophages^10^, therefore we aimed to determine whether IL-4Rα signaling on DCs is important in allergic disease of the airways by using CD11c^cre^IL-4Rα^−/lox^ mice. In the standard model of acute OVA-induced airway disease, mice are sensitized intraperitoneally with OVA and alum adjuvant, then challenge with OVA intranasally, and allergic airway disease is assessed 24 hours after the last challenge, when inflammation is highest^17^. In this model of acute OVA/alum induced allergic airway disease, we found no differences in airway resistance or elastance in CD11c^cre^IL-4Rα^−/lox^ mice after methacholine challenge (Fig. 1A). Allergic inflammation was also similar in CD11c^cre^IL-4Rα^−/lox^ mice compared to littermate controls, as seen by similar frequencies of eosinophils in bronchiolar lavage fluid and similar numbers of eosinophils in the lung, although neutrophils tended to be decreased in CD11c^cre^IL-4Rα^−/lox^ mice (Fig. 1B). Percentages of eosinophils and neutrophils are shown for all BAL samples, as actual numbers of cells obtained back from bronchoalveolar lavage may be less consistent. Interestingly, mice without IL-4Rα signaling on CD11c^+^ cells had significantly decreased levels of OVA-specific IgE compared to wildtype littermate controls, while levels of specific IgG1, IgG2a and IgG2b were similar (Fig. 1C). However, cytokines produced by restimulated draining lymph nodes were similar after both anti-CD3 and OVA restimulation (Fig. 1D). Mucus secretion in the airways was not affected by the loss of IL-4Rα signaling on CD11c^+^ cells (Fig. 1E,F).

**Figure 2:**
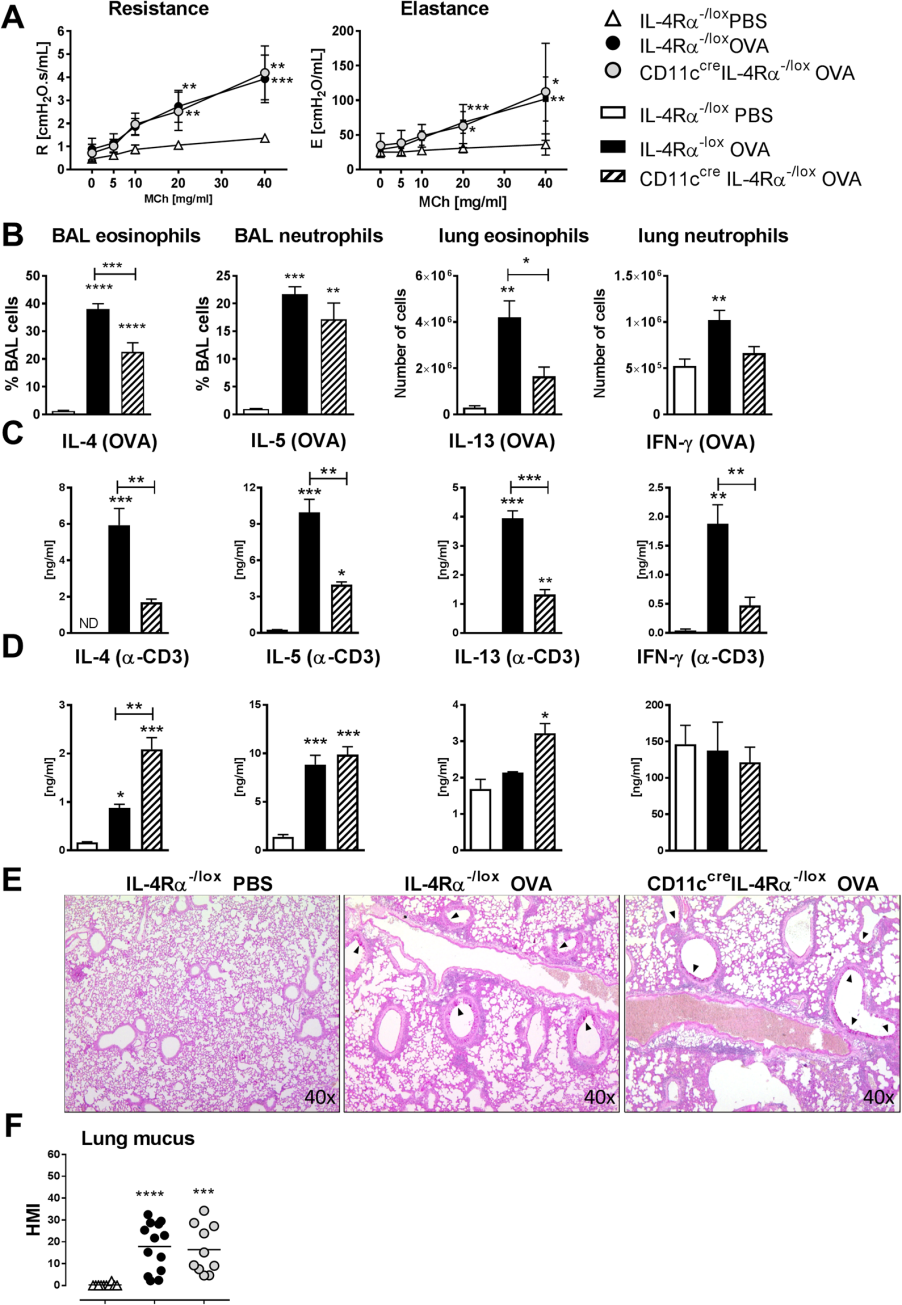
Allergic airway disease in CD11c^cre^IL-4Rα^−/lox^ mice in the OVA model without alum (day 24). (**A**) Airway resistance and elastance after methacholine challenge at day 21 in OVA-sensitized and challenged mice. (**B**) Eosinophils and neutrophils in the airways and lungs of challenged mice. (**C**) Cytokines measured by ELISA after restimulation of draining lymph node cells with OVA or (**D**) anti-CD3. (**E**) Mucus hypersecretion in the airways (100× magnification). (**F**) Goblet cell hyperplasia. HMI = histology mucous index. All graphs are representative of 2 experiments, with n = 5–6 per experiment. Significant differences are shown between IL-4Rα^−/lox^ PBS and the other groups unless otherwise indicated by brackets. *P < 0.05; **P < 0.01; ***P < 0.001.

In contrast, when alum adjuvant was omitted from the sensitization protocol, CD11c^cre^IL-4Rα^−/lox^ mice displayed attenuated disease compared to wildtype littermate controls, with similar AHR (Fig. 2A) but reduced eosinophils and neutrophils (Fig. 2B), suggesting that alum adjuvant may override the requirement for IL-4Rα signaling on DCs. Levels of both Th2 and Th1 cytokines were reduced in draining lymph node cells CD11c^cre^IL-4Rα^−/lox^ mice compared to wildtype controls after OVA restimulation (Fig. 2C), but not anti-CD3 restimulation (Fig. 2D), suggesting differences between general and antigen-specific responses. However, goblet cell hyperplasia was similar between wildtype and CD11c^cre^IL-4Rα^−/lox^ mice (Fig. 2E,F).

### Loss of IL-4Ra signaling on dendritic cells reduces house dust mite induced allergic airway disease

**Figure 3:**
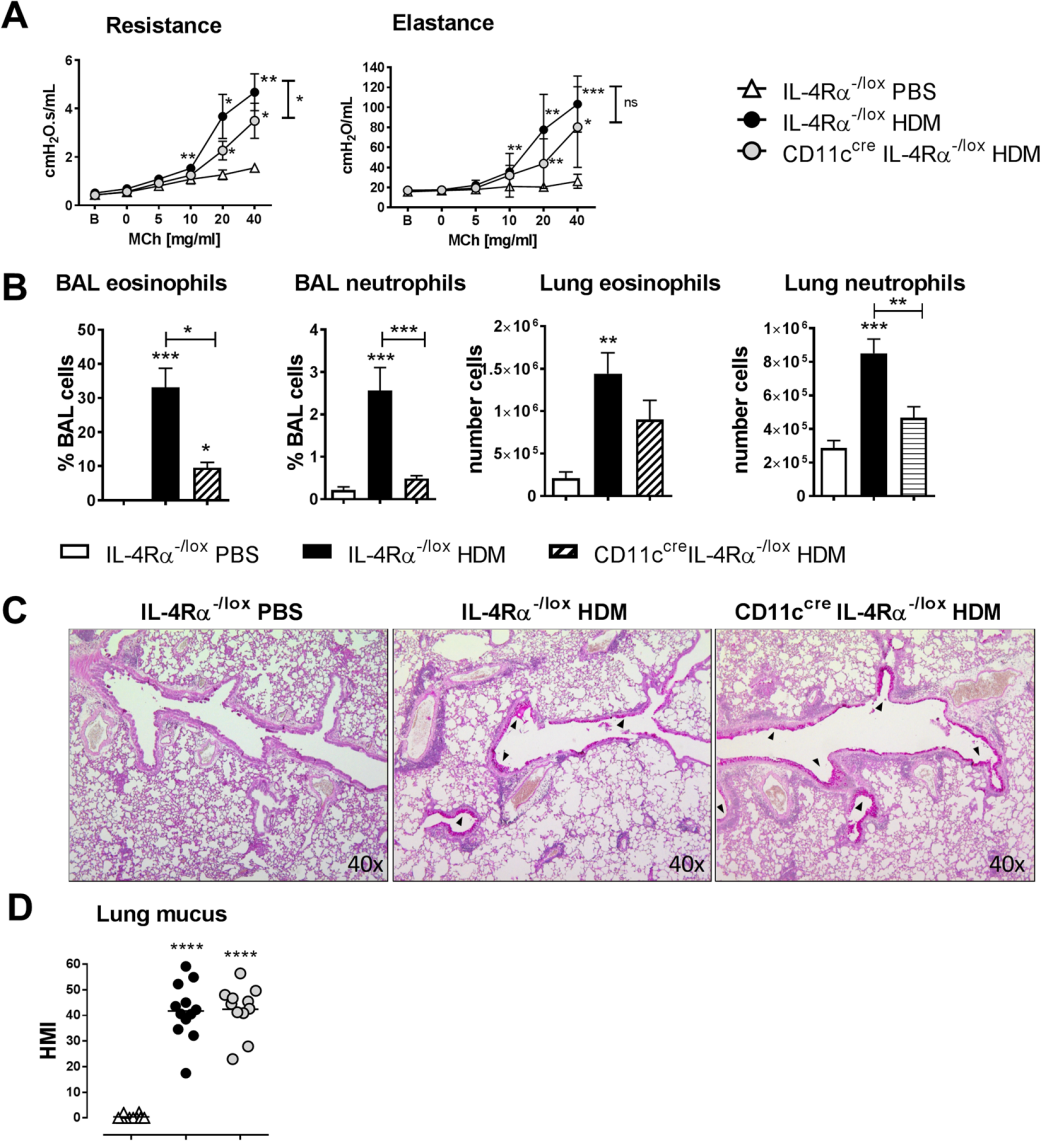
Allergic airway disease in CD11c^cre^IL-4Rα^−/lox^ mice in the house dust mite model (day 14). (**A**) Airway resistance and elastance after methacholine challenge in HDM-sensitized and challenged mice. (**B**) Eosinophils and neutrophils in the airways and lungs of challenged mice. (**C**) Mucus hypersecretion in the airways of challenged mice (100× magnification). (**D**) Goblet cell hyperplasia. HMI = histology mucous index. All graphs are representative of 2–3 experiments, with n = 5–6 per experiment. Significant differences are shown between IL-4Rα^−/lox^ PBS and the other groups unless otherwise indicated by brackets. *P < 0.05; **P < 0.01; ***P < 0.001.

Intranasal sensitization with house dust mite extract was subsequently used as a clinically relevant model of allergic airway disease that does not require adjuvant^17^. This model differs from the OVA model in that both sensitization and challenge are intranasal^1^. In the OVA model, inflammation is highest at 24 hours post challenge, whereas in the HDM model it peaks later, and starts to decrease 5 days after challenge^17^. Therefore, in the HDM model, allergic airway disease was assessed 3 days after the final challenge as previously described^1^. After HDM sensitization and challenge, CD11c^cre^IL-4Rα^−/lox^ mice showed significant protection against disease, displaying reduced AHR (Fig. 3A) and reduced eosinophils and neutrophils (Fig. 3B), but not goblet cell hyperplasia (Fig. 3C and D). CD11c^cre^IL-4Rα^−/lox^ mice lack IL-4Rα expression on both DCs and alveolar macrophages, but previously we examined responses to house dust mite in LysM^cre^IL-4Rα^−/lox^ mice, which lack IL-4Rα expression on macrophages, monocytes and neutrophils^18^, and found no significant difference in allergic airway disease, confirming that the attenuated responses seen in CD11c^cre^IL-4Rα^−lox^ mice were most likely due to abrogated IL-4Rα signaling on dendritic cells and not on alveolar macrophages.

### Reduced Th2 responses in the absence of IL-4Rα signaling on dendritic cells

**Figure 4:**
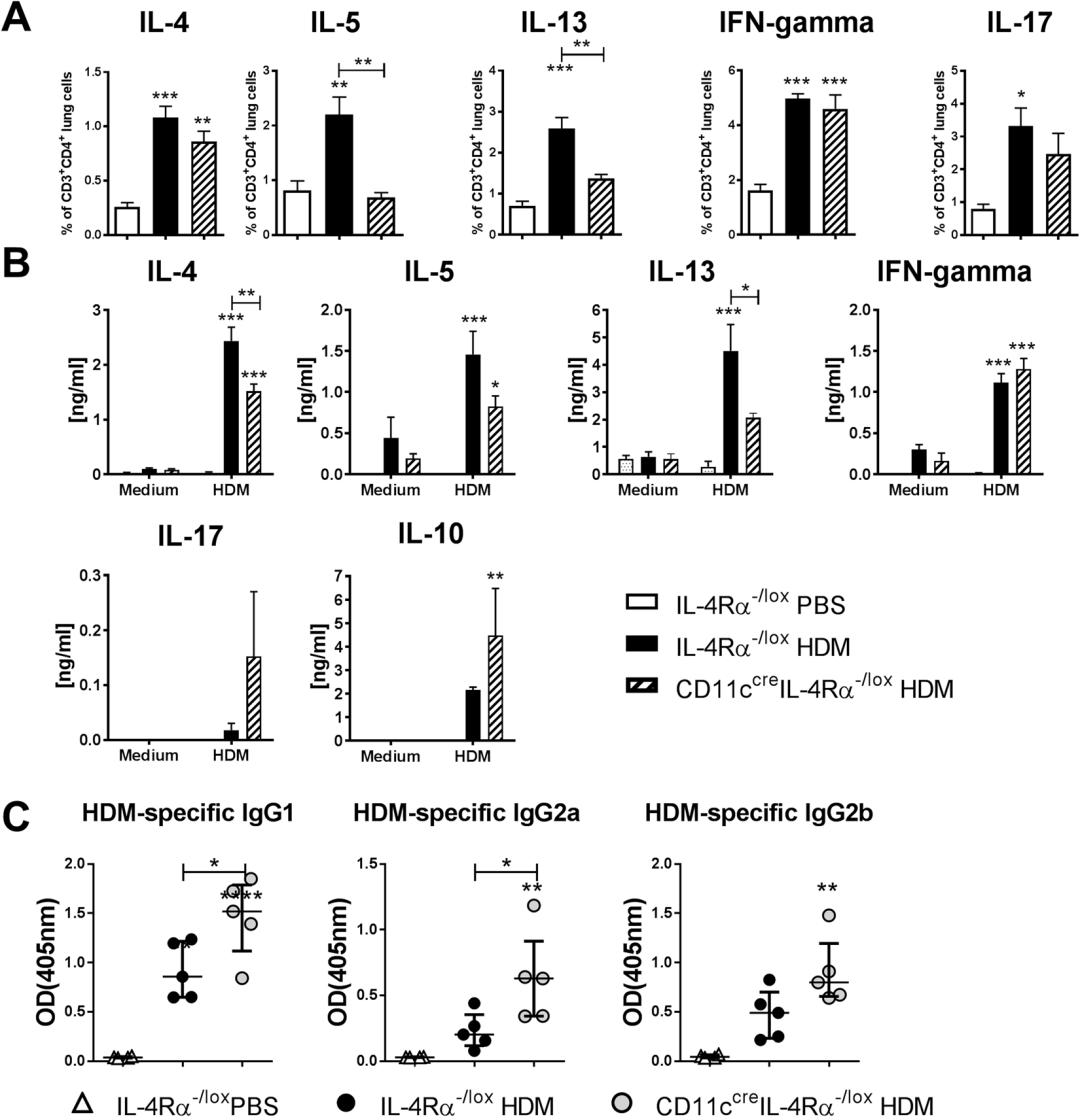
Reduced Th2 cytokine production in CD11c^cre^IL-4Rα^−/lox^ mice sensitized and challenged with HDM (day 14). (**A**) Cytokine production by lung CD4^+^ T cells after stimulation with PMA/ionomycin/monensin. (**B**) Cytokine production by mediastinal lymph node cells after restimulation with HDM extract, measured by ELISA. (**C**) HDM-specific antibodies measured in serum by ELISA. Graphs are representative of two experiments. Significant differences are shown between IL-4Rα^−/lox^ PBS and the other groups unless otherwise indicated by brackets. *P < 0.05; **P < 0.01; ***P < 0.001.

The observed reduction in allergic airway responses to HDM extract was accompanied by reduced Th2 cytokine production in both lung cells and mediastinal lymph node cells (Fig. 4A and B). Frequencies of CD4^+^ T cells producing Th2 cytokines from lungs were significantly reduced in CD11c^cre^IL-4Rα^−/lox^ mice (Fig. 4A), as were levels of Th2 cytokines induced by restimulation of mediastinal lymph node cells with HDM extract (Fig. 4B). Interestingly, there was a trend to increased HDM-specific IgG production in CD11c^cre^IL-4Rα^−/lox^ mice (Fig. 4C). We could not detect HDM-specific IgE, perhaps due to the short duration of the model (serum samples were collected at day 14).

**Figure 5:**
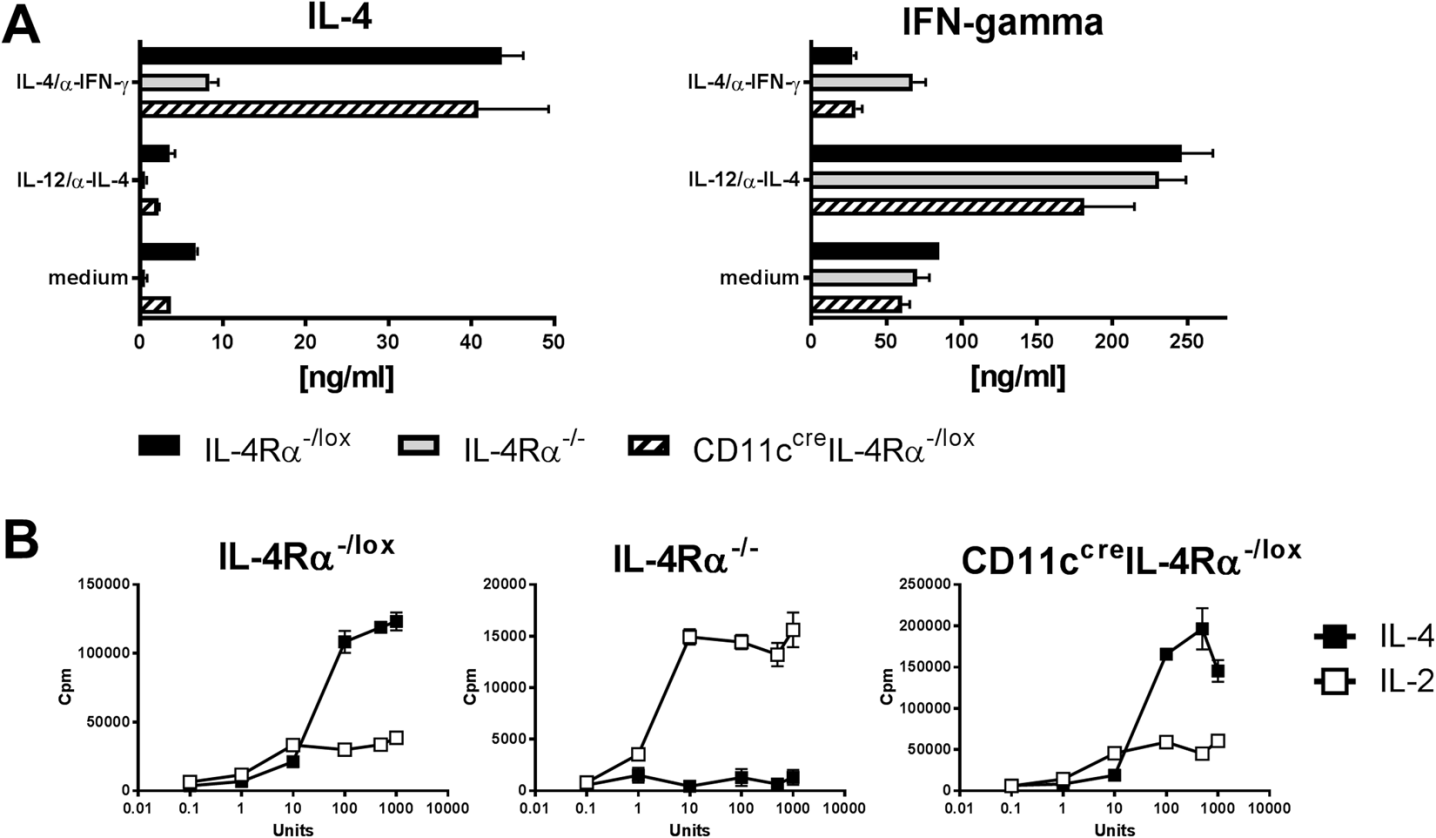
T cell responses in CD11c^cre^IL-4Rα^−/lox^ mice. (**A**) T cell differentiation assays. IL-4 and IFN-γ were measured in supernatants from purified CD4^+^ T cells of naïve mice stimulated with IL-4/anti-IFN-γ, IL-12/anti-IL-4 or medium as a control. (**B**) T cell proliferation assays. CD4^+^ cells from naïve mice were incubated with rIL-4 or rIL-2, and DNA synthesis was measured by thymidine incorporation for 18 hr. Graphs are representative of two experiments.

In order to ascertain whether T cells of CD11c^cre^IL-4Rα^−/lox^ mice could respond normally to IL-4, we performed differentiation and proliferation assays on purified CD3^+^ T cells from lymph nodes of naïve mice (Fig. 5A and B). T cells from CD11c^cre^IL-4Rα^−/lox^ mice showed similar capacity of cells from wildtype controls to differentiate into either Th2 or Th1 cells after exposure to IL-4/anti-IFN-γ and IL12/anti-IL-4, respectively, in contrast to T cells from IL-4Rα^−/−^ mice, which produced only low levels of IL-4 (Fig. 5A). T cells from CD11c^cre^IL-4Rα^−/lox^ mice were also able to proliferate in response to both IL-4 and IL-2 (Fig. 5B). Therefore, the decreased Th2 responses observed in the allergic airway disease model in CD11c^cre^IL-4Rα^−/lox^ mice were not due to any impairment of IL-4Rα signaling in their T cells.

## Discussion

Signaling through the IL-4Rα on DCs is known to play an important role in regulation of subsequent T cell responses very early in the immune response process^4,7,10^. Recently, we established a genetic mouse model by impairing IL-4Rα expression on DCs and alveolar macrophages, generating cell type specific CD11c^cre^IL-4Rα^−/lox^ mice^10^. We demonstrated that DCs and alveolar macrophages were efficiently impaired in IL-4Rα signaling. Using this mouse strain, we showed that impairment of IL-4Rα signaling on DCs in BALB/c mice resulted in hypersusceptibility to cutaneous leishmaniasis. We and others have previously found decreased IL-12 production by DCs in the absence of their ability to respond to IL-4, leading to decreased Th1 responses and classical macrophage activation^4,7,9,10^. In a study by Cook *et al*., injection of mice with recombinant IL-4, complexed with anti-IL-4mAb to ensure slow release, promoted an “alternative activation” phenotype, with upregulation of RELM-α and Ym1/2 on splenic DCs^4^. In addition, splenic and pleural cavity DCs from mice infected with *Schistosomas mansoni* or *Litomosoides sigmodontis*, Th2-promoting parasitic helminths, had increased RELM-α expression compared to naïve control mice^4^. Subsequently, DCs from *Retnla*^−/−^ mice (deficient in RELM-α) pulsed with SEA were adoptively transferred to naïve recipients. Mice that received *Retnla* ^−/−^ SEA DCs had reduced levels of IL-13 and IL-10 compared to those given WT SEA DCs, suggesting that DC production of RELM-α is required for optimal Th2 priming by helminth-activated DCs. The reduced ability of DCs to promote Th2 responses in the absence of IL-4Rα was not due to differences in antigen uptake, processing or presentation^4^. In the present study we further investigated the effect of IL-4Rα signaling on DCs in the initiation of allergic responses. Our results demonstrated attenuated allergic responses in CD11c^+^ specific IL-4Rα deficient mice in HDM-induced allergic airway disease.

Although CD11c^cre^IL-4Rα^−/lox^ mice lack IL-4Rα expression on both DCs and alveolar macrophages^10^, we previously found that impairment of IL-4 responsive macrophages had no significant influence on OVA- or HDM-induced allergic airway disease, using LysM^cre^IL-4Rα^−/lox^ mice established by our laboratory. While it has recently been reported that some inflammatory, monocyte-derived macrophages may still express IL-4Rα in LysM^cre^IL-4Rα^−/lox^ mice under certain conditions^22^, this does not appear to be the case for alveolar macrophages^18^. Therefore it is likely that the phenotypes observed in CD11c^cre^IL-4Rα^−/lox^ mice are caused by the loss of IL-4Rα expression on DCs.

Interestingly, the addition of alum adjuvant appeared to compensate for the loss of IL-4Rα signaling on DCs in OVA-induced allergic airway inflammation, perhaps by directly engaging the DCs and altering their activation phenotype^23–25^. It is known that alum can drive Th2 responses independently of signaling through the IL-4Rα^26^. Previously it was shown that antigen-specific T cells responses were initiated by monocytic DC-precursors in a NALP3-dependent mechanism in the OVA-alum model^24^. In an alum-free model using HDM sensitization and challenge, monocyte-derived DCs expressing FcεRI, termed inflammatory DCs, were found to be critical in the induction of allergic Th2 responses, and IL-4 produced by basophils seems to be required for this activation^1^. A more recent study also found DCs to be the target of alum, and showed that alum directly binds DC plasma membrane lipids, leading to increased antigen uptake, activation and more stable binding with CD4^+^ T cells^23^. In the absence of alum, both IL-4 and another pathogen/allergenic extract-derived signal seem to be required for optimal Th2 responses, such as protease activity or the ability to induce toll-like receptor or C-type lectin signaling^27–32^. Upon exposure to allergenic substances, both epithelial cells and DCs are capable of producing pro-Th2 cytokines such as IL-25, IL-33, TSLP and GM-CSF^32^.

The initiation of Th2 responses by DCs is not fully understood, and it is possible that more than one mechanism is involved^33–35^. CD11b^+^ DCs play a critical role in inducing Th2 responses^1,33–38^. Murine DCs can present antigen in an immunogenic or tolerogenic manner. Th2-promoting stimuli induce DC expression of the transcription factor interferon regulatory factor 4 (IRF4) in a subset of DCs involved in Th2 differentiation, known as programmed death ligand-2 (PDL2)^+^ DCs^33,34^. *In vitro*, PDL2^+^ DCs are promoted by IL-4 and inhibited by transforming growth factor-β (TGF-β)^33^. PDL2^+^ DCs could not induce differentiation of naïve T cells into Th2 *in vitro* or *in vivo*, but elicited strong Th2 responses in effector or memory CD4^+^ T cells^33^. Mice with IRF4 deficient CD11c^+^ cells had less secretion of IL-4, IL-15 and IL-13 after immunization with OVA and papain as well as during infection with the nematode *Nippostrongylus brasiliensis*. In an allergic airway disease model, deletion of IRF4 expression in CD11c^+^ cells attenuated Th2-type lung inflammation, and IRF4 was found to directly modulate IL-10 and IL-33 production by DCs, promoting Th2 differentiation and inflammation^34^. However, recently it was shown in a *S. mansoni* infection model that the reduction of Th2 responses in the absence of IRF4 in CD11c^+^ cells was due to impaired numbers of CD11b^+^ DCs carrying parasite antigens to the lymph nodes, and not due to the absence of expression of IRF4 related genes coding for OX40L, IL-10 and IL-33^35^. Therefore, IRF4 did not directly control the ability of intestinal DCs to polarise Th2 cells. Decreased DC migration and survival have both been suggested as reasons for the IRF4-dependent decrease in CD11b^+^ DCs^38,39^. IRF4 promotes both Th2 and Th17, and Kruppel-like factor 4 was required in IRF4-expressing DCs to promote Th2, but not Th17, responses *in vivo*^40^. CXCR5 expression on DCs is also important in Th2 immunity as it was required for colocalization of DCs and CD4^+^ T cells in the lymph nodes^41^. Promotion of Th2 responses was also partially dependent on PD1 and OX40 expression on T cells^33^.

How IL-4/IL-4Rα signaling on DCs affects DC function is incompletely understood. In several disease models in our laboratory we have observed that global IL-4Rα deficient mice appear to lack overall responsiveness to antigens, rather than a skewed Th1 type response, having smaller lymph nodes and weaker immune responses, as shown here in the house dust mite model of allergic airway disease. *In vitro*, IL-4 and IL-13 affect bone marrow-derived macrophage expression of MHCII and costimulatory molecules^8^. However, we and others have shown that the absence of IL-4Rα signaling on DCs does not affect expression of MHCII and classical costimulatory molecules such as CD86 and CD80 *in vivo*^4,10,42^. This supports previous suggestions that MHCII/CD80/CD86 expression cannot be used as a reliable proxy of DC ability to activate T cell responses, which is rather linked to their precise state of maturation and involves altered expression of many molecules^42^. Previously, IRF7^−/−^ DCs were found to produce elevated levels of IL-10 and decreased IL-12p70, impairing their ability to drive Th1 responses, despite increased levels of MHCII, CD80 and CD86^43^. Similarly, experiments with CD11c^cre^TGFβR2^lox/lox^ mice demonstrated that TGF-beta signaling to DCs was critically important in controlling autoimmune inflammation, but did not affect MHCII or costimulatory molecule expression^44^. Recently, it was found that PPAR-γ expression is required in CD11b^+^ DCs to induce potent Th2 effector responses in HDM-induced inflammation^45^. PPAR-γ expression enhanced DC migration to draining LN and Th2 priming capacity. As IL-4 promoted upregulation of PPAR-γ in lung resident CD11b^+^ DCs, these new data provide a clear link between IL-4Rα signaling on DCs and activation of Th2 responses. Furthermore, although we did not find an effect of IL-4Rα on migration of dendritic cells carrying antigens to the lymph nodes during priming (data not shown), DC IL-4Rα is likely to be important in the recruitment of memory Th2 cells to the lung, since IL-13, which signals primarily through the IL-4Rα ^2^, stimulates CD11b^+^CD103^−^IRF4^+^ lung DCs to produce the chemokine CCL17, which in turn recruits CCR4^+^ memory Th2 cells to the lungs^36^. ILC2s were shown to be an important source of this IL-13, and in their absence there was a dramatic reduction in Th2 cell numbers in the lungs after allergen challenge.

In summary, our study contributes to the understanding of DC regulation of Th2 responses by demonstrating that IL-4Rα signaling on CD11c^+^ cell plays a role in the generation of Th2 effector responses in allergic airway disease. Future studies of genes differentially regulated by IL-4Rα signaling on DCs may reveal pathways that could be targeted to inhibit Th2 responses or manipulated to favor activating or tolerogenic dendritic cell phenotypes.
