# Author Correction: House dust mite induced allergic airway disease is attenuated in CD11c^cre^IL-4Rα^−/l^°^x^ mice

**DOI:** 10.1038/s41598-018-23432-0

**Published:** 2018-04-11

**Authors:** Natalie Eva Nieuwenhuizen, Frank Kirstein, Jennifer Claire Hoving, Frank Brombacher

**Affiliations:** 1 International Centre for Genetic Engineering and Biotechnology (ICGEB), Cape Town component, University of Cape Town, Cape Town, South Africa; 2 Institute of Infectious Disease and Molecular Medicine (IDM), Division of Immunology, University of Cape Town and South African Medical Research Council (SAMRC), Cape Town, South Africa; 3 Present Address: Department of Immunology, Max Planck Institute for Infection Biology, Chariteplatz 1, 10117 Berlin, Germany; 4 Present Address: Biovac Institute, 15 Alexandra Rd, Cape Town, 7405 Ndabeni South Africa

Correction to: *Scientific Reports* 10.1038/s41598-017-19060-9, published online 17 January 2018

In the original version of this Article, there were errors in Affiliation 2 which was incorrectly listed as ‘Institute of Infectious Disease and Molecular Medicine (IDM), Division of Immunology, University of Cape Town, Cape Town, South Africa.’ The correct affiliation is listed below:

Institute of Infectious Disease and Molecular Medicine (IDM), Division of Immunology, University of Cape Town and South African Medical Research Council (SAMRC), Cape Town, South Africa.

In addition, the author Natalie Eva Nieuwenhuizen was incorrectly indexed.

These errors have now been corrected in the PDF and HTML versions of the Article.

